# A state-of-the-art review of tamoxifen as a potential therapeutic for duchenne muscular dystrophy

**DOI:** 10.3389/fphar.2022.1030785

**Published:** 2022-11-16

**Authors:** Valeria Botti, Olivier Menzel, Davide Staedler

**Affiliations:** ^1^ RE(ACT) Discovery Institute, C/O BLACKSWAN Foundation, Vuarrens, Switzerland; ^2^ Department of Biomedical Sciences, University of Lausanne, Lausanne, Switzerland

**Keywords:** tamoxifen, duchenne muscular dystrophy, estrogen, SERM, drug repurposing, DMD, rare diseases

## Abstract

**Introduction:** This systematic review analyzes the state-of-art repurposing of the drug tamoxifen (TAM) in the treatment of Duchenne Muscular Dystrophy (DMD), including its mechanism of action, toxicological findings, and past and ongoing clinical trials. A parallel aim of this work was to explore whether evidence exists to support further funding of investigation on TAM treatment for DMD patients with a pivotal trial in young patients. Bringing evidence and answering the scientific question of whether this treatment could improve the quality-of-life of DMD patients is needed to establish guidelines and accelerate access to promising therapies for DMD patients.

**Methods:** The search was conducted in January 2022 utilizing PubMed. All MeSH terms for “Duchenne Muscular Dystrophy” and “tamoxifen” were used. The inclusion and exclusion criteria were defined according to the PICOS framework.

**Results:** The included publications all explored the use of TAM with promising outcomes in muscular strength recovery and a decrease in pathology biomarkers. Two reviews recognize TAM as a potential treatment for DMD patients and state that drug repurposing plays a crucial role in the quest for a drug candidate to treat this rare disease.

**Conclusion:** According to available data, TAM shows promise as a treatment for DMD, both pharmacologically and clinically. However, published data to date are insufficient to definitively conclude the beneficial effect of TAM on quality-of-life and ultimately survival, particularly in the youngest patients diagnosed with DMD.

## Introduction

Duchenne Muscular Dystrophy (DMD) is an X-linked genetic disease that occurs in one out of 5,000 male patients. A mutation causes it in the dystrophin gene on chromosome Xp21.1. Patients suffer from progressive muscle weakness coupled with motor impairment (Mercuri and Muntoni, 2013), with progressive loss of ambulation and death usually occurring in the third or 4th decade of life. Diagnosis occurs at an average age of 4 ± 2.2 years (Magri et al., 2011).

A systematic review of the incidence and prevalence of the disease showed that DMD appears to be severe regardless of time assessed and location/country, with between 22% and 56% of patients likely to have lost ambulation and between 27% and 57% with cardiomyopathy (Ryder et al., 2017). It has been established that severity increases with age; the median age of 12 years was estimated for the loss of ambulation, while at age 20, patients are expected to start assisted ventilation.

The mortality of DMD is associated, in the advanced stages of the disease, with cardiac and respiratory problems. Indeed, as the disease progresses, patients develop respiratory failure and dilated cardiomyopathy, which will cause their death in a short time. Management of the disease in its more advanced stages has been markedly improved by the introduction, in early 1990s, of assisted nocturnal ventilation. Nocturnal ventilation was not initially received positively, because it was perceived as very impactful on quality of life and because of the absence of evidence on its relevance in terms of increased survival. However, a retrospective study by Eagle and others showed that the application of nocturnal ventilation since the 1990s has increased the survival rate of patients to 25 years by 53% (Eagle et al., 2022). Therefore, assisted ventilation has a central role in the overall care of DMD. In the management of DMD, significant results of increased overall survival have been obtained also by treating patients with cardioprotective drugs, in particular angiotensin-converting enzyme inhibitors (ACEi). It is known, in fact, that cardiomyopathies are responsible for the early death of at least 10% of patients, that the unfavorable evolution of cardiac conditions is inevitable with the increasing age of patients. The use of ACEi makes it possible to delay the consequences of cardiomyopathy, significantly reducing the hospitalization rate and lengthening the survival time (Porcher et al., 2021). Treatment with ACEi is also indicated as prophylaxis in the early stages of the disease, malignant and progressive myocardial involvement. These approaches make it possible to slow disease progression and increase patients’ hope and quality of life. However, there remains a need to evaluate and explore novel curative approaches, particularly those that can modulate the pathophysiology of the disease.

## Current strategies for DMD treatment

### Gene therapy

Gene therapy research is ongoing to provide a cure for DMD patients. The target is the mutant DMD gene, and treatments are aimed at increasing dystrophin production or analogs such as utrophin or alpha-7 integrin at physiological levels. Several isoforms of the dystrophin gene are expressed in muscle and non-muscle tissue because of alternative splicing occurrence from seven different promoters. This gene shows the highest known spontaneous mutation rate of any other human gene (Chamberlain and Chamberlain, 2017).

Molecular therapies with mutation-specific drugs, such as exon skipping oligonucleotides or small molecules promoting stop codon readthrough, are intrinsically limited to restricted DMD sub-populations. Furthermore, some patients may not be eligible for gene therapy because of pre-existing immunity to viral vectors and other reasons. Finally, both exon skipping and micro-dystrophin expression would be of relatively little help for all those patients in whom the amount of actual muscle tissue has already been drastically reduced. In these latter cases, a cell transplantation approach would be the ideal solution, but attempts in this direction have failed, and no clinical trials are currently ongoing. For all these reasons, there is a critical and pressing need to develop pharmacological therapies capable of addressing the numerous downstream consequences of dystrophin deficiency to improve quality-of-life and life expectancy in patients with DMD.

The European Medicines Agency (EMA) has given conditional marketing approval for ataluren (Translarna™), a small molecule designed to suppress the premature stop codon found in about 10% of patients (EMA, Accessed July 2022). Limits to this approach are the small number of patients that could benefit from this treatment, as it targets only patients with a non-sense mutation of the dystrophin gene, and only approximately 13% of patients with DMD present with this nonsense mutation (Kent et al., 2005).

Another therapeutic strategy uses antisense oligonucleotides (AONs) to restore the disrupted reading frame and produce a shorter version of dystrophin protein. Eteplirsen, used for patients with a confirmed mutation of the DMD gene amenable to exon 51 skipping, has significantly slowed disease progression. However, repeated infusions are required as its effect is transient, having pre-mRNA as a target. A limitation of this treatment is that the exon 51 skipping genetic population of DMD patients represents only about 13% of all DMD patients (McDonald et al., 2021).

Golodirsen, a similar AON that targets exon 53, has shown an increase in dystrophin production levels in a first-in-human clinical trial (NCT02310906). However, similar to eteplirsen, the proportion of DMD patients with out-of-frame deletions amenable to exon 53 skipping is small, only approximately 8% (Frank et al., 2020).

Over the years, remarkable efforts have been made toward developing therapies that replace or repair the defective dystrophin gene and permit the production of dystrophin analogs.

New gene correction approaches *via* genome editing show a great potential for the treatment of DMD. These approaches are based on CRISPR/Cas9 delivered *via* viral vectors (Mendell and Rodino-Klapac, 2016). CRISPR/Cas nine technology in particular allows the DMD gene to be corrected *via* intragenic DNA deletions or excisions or even *via* similar strategies with exon skipping at the DNA level by introducing antisense oligonucleotides. Other strategies, for example, removal of duplicated exons, precise correction of causative mutations, and induction of expression of compensatory proteins such as utrophin, can be implemented using CRISPR/Cas9 technology. To date, more than 25 studies have been completed and published. These are studies at the pre-clinical stage, but overall they offer a clear view of the great potential of this technique as a future support for gene therapy for DMD (Mendell and Rodino-Klapac, 2016; Mollanoori et al., 2021).

Nonetheless, technologic, cost and safety issues obstruct the development of these approaches. An analysis conducted by Shukla et al., in 2019 showed that gene therapies are among the most expensive treatments available. According to their analysis, healthcare systems are not prepared to assume the cost of future treatments for various rare diseases and common diseases of epidemic proportions. In this context, the potential and developments in the application of CRISPR/Cas9 technology could lead to a reduction in the cost of these types of therapies, enabling greater sustainability for the health care system and democratization consequences, benefiting a growing number of patients.

Also, indirect costs are a significant feature of DMD and should play a role in informing appropriate care and coordinated financial planning of health and social care. Per capita cost burden increases with disease progression, and people seem to live longer with the condition. This is attributed to the widespread prescribing of corticosteroids, improved access to ventilation, and the publication of more thorough and specific guidelines of care. This should also be considered in determining costs associated with supporting and treating DMD patients. (Ryder et al., 2017).

### Glucocorticoids

The current standard-of-care treatment for DMD patients is glucocorticoids (GCs), which counteract inflammatory responses at the myocyte level. GCs bind to the glucocorticoid receptor (GR) in the cytoplasm, whereas the main transcriptional isoforms are GRα and GRβ. When no ligand is binding, GR is free in the cytoplasm and is chaperoned by two heat shock proteins 90 (Hsp90) molecules, by Hsp90-binding protein 23 (p23), Hsp70 and immunophilins, that protect it together from degradation and make it ready for binding with substrates. When a substrate binds to the GR, Hsps get released, and GR is free to migrate to the nucleus of the myocyte and back, creating its own interactome. When GCs bind to GR, a series of physiological consequences that can be classified as genomic and non-genomic actions happen. One of the main targets of the GR is the inhibition of NF-kB, which is the major activator of gene-coding for pro-inflammatory cytokines, chemokines, and immunoreceptors, along with other critical biochemical players inhibited by GCs. When the complex GC-GR translocates to the nucleus, the binding to gene transcription leads to expression changes in around 10–20% of the genes. On the other hand, non-genomic actions give a much steadier response and consist of the activation of Toll-like receptors (TLRs), p38 mitogen-activated protein kinases (MAPKs), c-Jun N-terminal kinases (JNKs), the NF-κB and activator protein 1 (AP-1), the Janus kinase/signal transducer and activator of transcription proteins (JAK/STAT), and transforming growth factor-β (TGF-β). In MDX mice, these pathways are activated to increase the expression of α7 integrin and compensate for the loss of dystrophin.

Very recently, a 3-year randomized clinical trial, presented by Guglieri and co-authors, showed that daily treatment with prednisone or deflazacort, compared with intermittent treatment with prednisone (10 days on and 10 days off), resulted in significant and overall improvement of a composite outcome that included measures of motor function, lung function, and treatment satisfaction. The results of this suggest that the use of a daily corticosteroid regimen, compared with the intermittent regimen, characterize superior clinical outcomes as the initial treatment for boys with DMD (Guglieri et al., 2022).

However, treatment with GC (prednisone and deflazacort) has been shown to have several side effects for DMD patients, including hyperglycemia, delayed growth, osteoporosis, cataracts, and suppression of the immune system, potentially leading to life-threatening infections. Steroid-sparing treatments that allow for a dose reduction of synthetic GC need to be considered to improve patients’ quality-of-life (Herbelet et al., 2020).

### Emerging therapies

The current landscape of drug development is targeting different molecular mechanisms involved in the pathology of DMD to explore the efficacy of several pathway-interferent drugs.

In the context of the upregulation of utrophin, a dystrophin paralogue, a drug called Ezutromid (SMTC1100) has been identified *via* high-throughput screening. This compound was developed by Summit Therapeutics and tested up to a phase 2 clinical trial (NCT02858362) but was terminated due to lack of efficacy and a too challenging pharmacokinetic profile (Tinsley et al., 2015; Muntoni et al., 2019). Another aryl-hydrocarbon antagonist (SMT022357), of second generation, was developed and *in vivo* results in mdx mice from a study of 2015 showed a 47% decrease of serum Creatine Kinase (CK) levels (Guiraud et al., 2015) (May) and a 1.7- and 1.3-foldincrease of β-dystroglycan and dystrobrevin expression, respectively. This data is promising towards a potential improvement of muscle membrane stability in DMD patients when utrophin acts as a dystrophin surrogate.

Myostatin, a secreted growth differentiation factor, is known to have a role in DMD pathogenesis. Several clinical studies have tried to witness a translation of beneficial pre-clinical data to a clinical improvement, but many did not succeed (Rybalka et al., 2020).

In this context, CMV. huFollistatin344, which is an intramuscular transfer of the human follistatin, a protein that binds to myostatin and inhibits its activity, was assessed for efficacy in a phase 1-2 clinical study (NCT02354781) in Becker muscular dystrophy (BMD) patients. Results showed an excellent safety profile that mirrored preclinical findings, as well as an improvement in the distance walked following injection of the quadriceps muscles (Mendell et al., 2015). Limitation to this application are that its use on DMD patients is still to fully investigate, since pathogenesis are different and symptoms of BMD are lighter.

The management of myostatin pathway was also modulate by means of the oral histone deacetylase inhibitor Givinostat, that has the potential to increase expression of follistatin *via* epigenetic control. Givinostat was assessed in a randomised, placebo-controlled phase 3 trial (NCT02851797). According to non-peer-reviewed data conference in 2022, the trial met the primary endpoint, the change in 4SC, at 18 months of treatment in the group of patients with baseline vastus lateralis muscle fat fraction in the range of >5% and ≤30% (Businesswire, Accessed October 2022).

Further studies have considered the application of Idebenone, a synthetic short-chain benzoquinone derivative acting as a transporter in the electron transport chain of mitochondria, for the treatment of DMD. In 2011, Buyse and co-workers showed that Idebenone, was successful in addressing the muscle cell injury that occurs in dystrophin deficiency (Buyse et al., 2011).

In 2019, following the afore-mentioned result of the candidate, a retrospective cohort clinical study with Idebenone was published. Data showed that, on 18 DMD patients that were given 900 mg/day of Idebenone and were not using GCs, the experimental drug improved respiratory function (Servais et al., 2019). However, the limited size of the study does not permit robust conclusions from the statistical profile.

More recently, anti-inflammatoriy drugs were also considered and, in 2020, an open-label study phase IIa using steroidal anti-inflammatory Vamorolone investigated efficacy and safety of this compound in DMD patients for 18 months. Vamorolone-treated participants showed significant improvement in the run/walk 10 m velocity test and climb four stairs velocity test compared to corticosteroid-naïve matched patients. Time to stand velocity did not show any difference in the comparison of the two groups. The trial design for this test was too weak itself to prove an important benefit compared to GCs, and further research with a blinded study designed might be needed. (Smith et al., 2020).

Between 2013 and 2016 a drug combination was investigated to find an effective treatment for Duchenne patients. At University Children’s Hospital of Basel, a combination of l-Citrulline and Metformine was tested in a randomized double-blind placebo-controlled clinical trial on 47 ambulant patients. In this study, remarkable are both the sample size and the study design, based on the knowledge that nitric oxide precursors play a role in muscle protection and its correct metabolism in patients with DMD. The study did not show any remarkable difference between treatment and control arm (Hafner et al., 2019).

Lastly, since a key feature of DMD is the widespread inflammation due to chronic activation of the NF-κB signaling pathway, this last segment has also been in the focus of scientists developing potential new treatment agents. In this context, NBD (NEMO binding domain), a specific inhibitor peptide of NF-κB signaling pathway, has shown to reduce skeletal muscle damage and increase muscle function in mdx mouse. NBD is currently under investigation moving forward to clinical trials after additional efficacy and safety pre-clinical studies. (Messina et al., 2006; Peterson et al., 2011; Delfín et al., 2011).

The important variety of investigative approaches to treating DMD show the extent to which there is a need to develop an effective treatment for this disease but also a need to better understand its pathophysiological mechanisms.

## Tamoxifen as a potential therapeutic

In this review, we investigate the state-of-art tamoxifen (TAM) repurposing for treating DMD. The story of the development of tamoxifen as a pioneering medicine in cancer treatment originates in the confluence of two research paths. The first sought to understand why only some women who developed breast cancer responded to endocrine therapies. The second led to the chance discovery of nonsteroidal anti-estrogens.

The team of reproductive endocrinologists at Imperial Clinical Industries (ICI) Pharmaceuticals Division (now AstraZeneca) discovered the compound ICI46,474 (Harper and Walpole, 1967), which became tamoxifen. TAM is the pure trans isomer eventually developed for ovulation induction in subfertile women and the treatment of breast cancer in elderly women (Jordan 2006).

TAM is a small lipophilic molecule belonging to the selective estrogen receptor molecule (SERM) class, part of the SER modulators (SEM) family.

As a pro-drug metabolized in the liver predominantly by CYP2D6, it gets metabolically activated to 4-hydroxytamoxifen, or alternatively *via* N-desmethyltamoxifen to 4-hydroxy-N-desmethyltamoxifen. The hydroxyl metabolites of TAM have a high binding affinity for the estrogen receptors (ERs) (Jordan 2007).

The physiological effects of estrogen are manifested through two ER isoforms, ERα and ERβ. These two variants are encoded on different chromosomes and belong to the superfamily of steroid receptors; therefore, they have partial structural homology but remain quite different in ligand binding affinity. The ratio of ERα: ERβ in a target tissue can be used to investigate target site modulation. A high ERα: ERβ ratio is indicative of high levels of cellular proliferation, whereas a predominance of ERβ over ERα correlates with inhibition of proliferation (Maximov et al., 2013).

TAM has been used for over 20 years in treating breast cancer, where its mechanism of action is well known, as well as the clinical outcomes in women. SERM molecules act as mimetic ligands or antagonists on ERs (Jordan 2007).

Most of the effects of estrogen, TAM, and TAM metabolites result from their high-affinity binding to ERα and Erβ, which are expressed in estrogen-responsive tissues of both males and females, including skeletal muscle. Several ERβ isoforms exist. ERβ1 is considered the physiologically active isoform, whereas ERβ2, a longer isoform with a much-reduced affinity for estrogens, acts in a dominant negative manner for the other ERs.

Expression of ERβ has been found in muscle fibers and capillaries in humans, and, in men and post-menopausal women, estrogens are produced predominantly in muscles. The main physiological effects of estrogen in skeletal muscles are to increase force output and sustain muscle recovery from disuse atrophy after induced injury.

The presence of the ERβ receptors in muscle fibers and capillaries is crucial in the physiological response to ER-mediated transcriptional activity and the consequent biological effects. One mechanism of action is thought to be, in the muscle, an enhancement of myogenesis and angiogenesis mediated by the vascular endothelial growth factor (VEGF). Such ER-mediated effects may favor muscle tissue repair and adaptation after training (Wiik et al., 2005). Estrogen receptors regulate gene transcription through classic estrogen response elements (EREs) and AP-1 responsive genes. Tamoxifen acts as an ER antagonist on EREs but as ERβ agonists/partial agonists on activator protein 1 (AP-1) responsive genes, which are responsible for several functions in cell growth and proliferation (Jain and Koh 2010).

It has been proven that TAM operates as a radical scavenger and a cytosolic calcium modulator, inhibiting muscular fibrosis (Vitiello et al., 2019). The mechanisms underlining the modulation of calcium cellular signaling by TAM have not yet been fully elucidated. It is known that estradiol can also bind G protein-coupled estrogen receptor 1 (GPER1), inducing rapid protein kinase mediated signaling. This has a direct impact on calcium signaling, *via* cAMP production and subsequent Ca2+ mobilization. In this context, TAM may act as an agonist of GPER1 and thus directly affect calcium signaling (Birnbaum et al., 2022).

Research on TAM use for breast cancer suggests a genetic pathway may also be involved. It has been shown that in addition to the antagonistic, partial agonist/antagonistic, or even complete agonist activities of TAM on genes regulated by estradiol (E2), TAM is also capable of regulating the expression of some genes that are not regulated by either E2 or other SERMs. Like the other categories of TAM-modulated gene expression, this regulation is mediated *via* the ER (Frasor et al., 2006).

## Systematic review methodology

This analysis aims to provide clarity as a systematic review of the state-of-art TAM repurposing in DMD.

The information sources and search strategies were designed in collaboration with the University of Geneva, Switzerland, to include all PICOS characteristics combined through Boolean variables. The lead author conducted the final search over PubMed on the 13th of February 2022. All MeSH terms for “Duchenne Muscular Dystrophy” and “Tamoxifen” were used. An advanced search was carried out for DMD MeSH terms within text together with Tamoxifen MeSH terms within the text. The European Medicines Agency website was also consulted for this review. PRISMA guidelines (Page et al., 2021) were followed. The inclusion and exclusion criteria were defined according to the PICOS framework. The review was not registered on an International Prospective Register of Systematic Reviews as it includes studies with different populations (mouse models and humans).

## Results

For the systematic review of TAM, 15 publications were identified and screened in Pubmed, dating from 1996 to 2021.Two reviews were removed due to duplication, and six animal studies were excluded because they were not classified as pertinent (i.e., they were gene ablation studies). One cohort study was excluded as not pertinent to pathology. One clinical trial protocol was included, but the results are not yet published. Five publications were included: one public summary of opinion, three pharmacological studies *in-vivo*, and 1 Phase 1 randomized clinical trial (RCT).

A first study conducted by Koot et al., in 1991 introduced the use of TAM to characterize the effect of estradiol on exercise-related creatine kinase from skeletal muscle. They showed that TAM induced protection of rat skeletal muscles from harmful contractions after long-term treatment, while short-term (24 h) treatment had no efficacy.

Later, given the urge to find a therapeutic remedy for DMD, the research group of Dorchies et al. at the University of Geneva in Switzerland conducted a pioneer study in repurposing TAM in *mdx* ^5Cv^ mice, a commonly used DMD mouse model.

In this study, the authors found that TAM improves muscle force, diaphragm, and cardiac structure in *mdx* ^5Cv^ mice. Interestingly, the estrogen receptors ERα and ERβ were several times more abundant in dystrophic than in normal muscles, and TAM normalized the relative abundance of ERβ isoforms (Dorchies et al., 2013; Gayi et al., 2018a).

TAM also reduced fibrosis in the diaphragm while increasing its thickness, myofiber count, and myofiber diameter, thereby augmenting by 72% the amount of contractile tissue available for respiratory function. After TAM treatment, they showed that the development of fibrosis in dystrophic hearts had decreased by ∼53%.

Administered orally for 15 months at 10 mg/kg/day to *mdx* ^5Cv^ mice, TAM caused remarkable muscular improvements as demonstrated by the mice’s grip in the wire test, showing how body musculature developed greater strength. The triceps surae, a group of muscles in the leg, showed an evident enhancement of contractile features; the diaphragm, the most severely affected muscle in dystrophic mice, was substantially thicker with reduced fibrosis; the heart also showed a significant reduction in the extent of fibrosis (Dorchies et al., 2013).

Following the research from M. Dorchies, TAM was granted orphan designation from the EMA on 12 October 2017, for the treatment of DMD (European Medicines Agency 2017).

The encouraging results from previous studies, in particular in animal models, led to a single-arm monocentric phase I trial (NCT02835079) conducted at the Hebrew University of Jerusalem, Israel (Tsabari et al., 2021). This study showed that orally administered TAM treatment at a dose of 20 mg/day retained muscle and respiratory function compared with a significant deterioration of age-matched historical control patients*.* Patients were treated for up to 3 years; the only secondary effect noted was mild gynecomastia*.* Through the trial, tamoxifen treatment resulted in a statistically significant decline in the creatine phosphokinase biomarker in treated patients, in contrast with the pathology-related high levels, which are a clear indicator of tissue damage and oxidative stress. Pulmonary function, tested by spirometry, showed stable lung function throughout the trial period for all TAM-treated patients, compared to a statistically significant function decline in the matching historical group. Moreover, statistical significance was found in the motor function test, 6MWT (6 m Walking Test), and NSAA (Northstar Ambulatory Assessment) for the TAM group, in which parameters were stable in contrast to the historical group, where deterioration of muscle-related functions was observed. The main limitations of this study were the absence of a placebo control arm and the inclusion instead of historical data comprised of patients with 1 year of treatment with GC. Additionally, the study included only a small number of patients, of which only eight were appropriate for statistical analysis. However, these results undoubtedly encourage further research as this study proved a safety profile in patients with DMD and stabilization of motor functions.

The use of TAM has also been explored in the X-linked myotubular myopathy (XLMTM), showing that XLMTM is an interesting model of neuromuscular genetic disease for the understanding of the mechanism of action of TAM (Maani et al., 2018). This rare muscular congenital disorder results in death within the first 2 years of life. Mutations cause it in the myotubularin (*MTM1*) gene. In a study in mice models (*Mtm1*-KO) with X-linked myotubular myopathy, Maani and co-workers showed significantly prolonged survival and enhancement of muscle strength following treatment with TAM. Additionally, the authors showed that treating *Mtm1*-KO mice with estradiol, a pure estrogen receptor agonist, daily (*via* subcutaneous injection) significantly improved survival compared to untreated *Mtm1*-KO mice. Estradiol-treated mice were also more active and subjectively stronger than placebo-treated *Mtm1*-KO mice. This result suggests that TAM’s potential mechanism of action is through an estrogen agonism pathway. TAM’s complete mechanism of action in XLMTM has not yet been elucidated. Still, researchers hypothesized that alternative pathways and compensatory mechanisms ameliorate dystrophic muscle function and force output, possibly to levels above wild-type muscle.

Another study utilizing the XLMTM mouse model, carried out by Gayi and co-workers, explored TAM’s ability to restore physiological parameters of XLMTM (Gayi et al., 2018b). In this protocol, TAM was orally administered in *Mtm1*-deficient mice beginning shortly after weaning. The authors showed an overall improvement in the health state of the animals. In particular, they showed a body strength and muscle structure increase in conjunction with normalization of molecular markers of the disease; furthermore, excitation-contraction coupling of muscle fiber amelioration and a diaphragm size increase of 2-fold was observed. After the treatment, levels of DHPR (dihydropyridine receptors), an L-type Ca2+ channel that regulates excitation-contraction coupling, were normalized to the physiological level.

In wild-type skeletal muscle, DHPRs in the transverse-tubular membrane contribute to Ca2+ influx during prolonged muscle activation or store depletion.

Table 1 provides details of the six publications included where TAM was explored in DMD or similar models of muscular pathology. Criteria of inclusion were the use of TAM in a patient population of young males or muscular dystrophies animal models (Duchenne or X-linked Myotubular Myopathy). X linked myotubular myopathy has been included in Table 1 as a recognized model for evaluation and validation of treatments for DMD. Studies on women have been excluded. Despite the different populations and diseases evaluated, clinical outcomes inherent to TAM’s effect on the striated muscle are relevant to the DMD population.

**TABLE 1:** Summary of studies Evaluating TAM in DMD and XLMT.

Authors	Age and Target	Muscular Pathology	Treatment Duration	Dosage of TAM	Sample Size	Clinical or Biochemical Improvement after TAM Administration	Statistical Significance
**Koot et al. (1991)**	Three weeks old; male and female **WISTAR rats**	No pathology; muscle physiology experiment	3 weeks	1) 0.25, 0.50, 1.00 mg/kg/day to female rats (Exp.1)	1) *n* = 3	1) Yes; total amount of CK released during the experiment was markedly lower for TAM treated mice vs controls	1) *p* = 0.012
				2) 5.46 mg/kg/day to female rats after ovariectomy (OVX) (Exp. 2)	2) *n* = 16	2) Yes; OVX resulted in an increased cumulative CK release. Administration of estradiol (E2) restored the level of CK release even below the values of untreated rats. Administration of TAM to OVX mice reduced the levels of CK release even lower than E2. The lowest level of CK was achieved by injecting both TAM and E2	2) *p* < 0.002
				3) 0.5 mg/kg/day to male rats (Exp. 3)	3) n = 3	3)The cumulative CK efflux from soleus muscles was significantly lower compared to the control group	3) *p* < 0.002
**Dorchies et al. (2013)**	From 3 weeks of age; **mdx** ^**5Cv**^ **mouse model**	DMD	15 months	10 mg/kg/day	n = 14 males (Dys Control); n = 12 males (Dys + TAM); n = 12 (wt Control)	1) Yes; TAM stabilized the ERb2-to-ERb1 mRNA ratio closer to Wt Control levels	TAM compared to Dys (untreated) group:
							1) *p* ≤ 0.001
						2) Yes; TAM enhanced the accumulation of several structural proteins, such as the dystrophin homologue utrophin, a7 integrin, and alphaB-crystallin compared to Wt Control	2) *p* ≤ 0.001
						3) Yes; TAM prevented the development of fibrosis in dystrophic hearts by ∼ 53% compared to Dys Control group	3) *p* ≤ 0.01
						4) Yes; TAM augmented the amount of contractile tissue in the DIA by 72% (diaphragm thickness, fiber count, fiber layers) compared to Dys Control group	4) *p* ≤ 0.001
						5) Yes; TAM had positive effects on the wire test score, increasing the time in which the animal was able to keep their grip compared to the Dys Control Group	5) *p* ≤ 0.001
**Maani et al. (2018)**	At birth; **Mtm1 KO mice**	Myotubular Myopathy	1.1) 27 days	a) High-dose 1.1) High-dose starting at 21 days from birth1.2) High-dose starting at 30 days from birth b) Low-dose b.1) Low-dose starting at 14 days from birth b.2) Low-dose starting at 21 days from birth b.3) Low-dose starting at 30 days from birth	a) n = 6	a) Yes; higher grip strength compared to control	a) *p* < 0.0001
			1.2) 36 days		1.1) *n* = 20	1.1) Yes; improved survival compared to control	1.1) *p* = 0.05
			2.1) 14 days		1.2) *n* = 5 (subset) b) *n* = 9 2.1) n = 9	1.2) No; improved survival only in the subset of treated animals surviving beyond the first 2 days of treatment b) Yes; higher grip strength compared to control (b.1, b.2, b.3) Yes; improved survival compared to control	1.2) *p* = 0.03 (subset) b) *p* < 0.0001
			2.2) 36 days		2.2) *n* = 14		2.1) p<=0.001
			2.3) 24 days		2.3) *n* = 8		2.2) *p* = 3,1 × 10^-5
							2.3) *p* = 0.04
**Gayi et al. (2018a)**	1) 23 ± 1 day	X-linked myotubular myopathy	From weaning, life-long	1), 2) a) 30 mg per kg/diet (actual intake 4, 5, 6 mg/kg day)	1) N = 8–18 mice	1) All doses of TAM extended the life span of Mtm1^-/y mice compared to control	1) N/A
	2) 42, 84, 210 days			b) 3 mg per kg/diet	2) *n* = 7 triceps per group	TAM preserved motor function of Mtm1^-/y^ mice close to wt	2) Absolute phasing tension overtime increase with TAM in XLMTM mice compared to untreated XLMTM *p* ≤ 0.05. Specific phasing tension overtime increase with TAM in XLMTM mice compared to untreated XLMTM *p* ≤ 0.01. Absolute tetanic tension overtime increase with TAM in XLMTM mice compared to untreated XLMTM *p* ≤ 0.05. Specific tetanic tension overtime increase with TAM in XLMTM mice compared to untreated XLMTM *p* ≤ 0.001
				c) 0.3 mg per kg/diet		2) TAM significantly enhanced the force (tension) in Mtm1^−/y^ mice. Specific phasic force of Mtm1^−/y^ mice increased with TAM at all examined ages. Specific force of Mtm1^−/y^ mice markedly augmented with TAM at all stimulation frequencies	
**Tsabari et al. (2021)**	6–14 years;	DMD	3 years	20 mg/day	*n* = 13 ambulant boys	1) Yes; significant decline of CPK	1) A statistically significant reduction in levels is seen between baseline and 6, 9, 18, 24, 30 and 36 months
	**Human subjects**					2) Stabilization of lung function compared to matched historical control (n = 88)	2) Statistically significant
						3) Stabilization of motor function tests 6MWT and NSAA compared to historical groups	3) Significant amelioration but no statistical evidence; in contrast, rapid decline of motor functions of historic control group is statistically significant
**Nagy et al. (2019)**	6.5–12 years (Group A), 10–16 years (Group B)	DMD	48 weeks	20 mg daily (placebo controlled randomized, double-blind efficacy and safety trial)	Group A: 71 ambulant patients	To be published	N/A
	**Human subjects**				Group B. 20 non-ambulant patients		

**Abbreviations:** mg: milligrams; kg: kilogram; CK:creatinine phosphokinase; OVX:ovariectomy; E2:estrodial; TAM: tamoxifen; Erb2 and Erb1:estrogen receptors; wt:wild type; DIA: diaphragm; dys:dystrophines.e.; standard error of the mean; 6MWT:6 min walk distance; NSAA:north star assessment; N/A:not applicable.

## Discussion

The potential of the use of TAM for the treatment of DMD has been known for 2 decades. Several studies have shown the musically beneficial effects related to the administration of TAM and its action on estrogen receptors. In particular, the study conducted by Dorchies et co-authors, showed surprising results, in which the application of TAM made dystrophic muscles even stronger than wild-type muscles (Dorchies et al., 2013). This suggests that the mechanism of action does not involve the restoration of the dystrophin-mediated signaling pathway but somewhat alternative paths and compensatory mechanisms that act in synergy toward improving dystrophic muscular function and force output even above an unexercised normal muscle level.

The study also established that TAM treatment leads to an accumulation of structural proteins. One of them is utrophin, a dystrophin homolog proven to be of therapeutic interest, acting as a surrogate of the missing dystrophin.

In fact, since the cloning of the utrophin gene, several observations have led to the hypothesis that utrophin may functionally compensate for dystrophin deficiency and perform complementing roles in normal functional or developmental pathways in muscle. There is a high degree of similarity between utrophin and dystrophin, especially regarding functionally essential protein domains and binding partners in muscle. Additionally, utrophin is up-regulated during periods with a lack of necrosis in dystrophin-deficient muscle (Perkins and Davies 2002).

In parallel to studies on DMD, composites outcomes form studies conducted on XLMTM can help to further elucidate the pharmacological effects of TAM in DMD, as all these parameters play a key role in regulating muscular function, even if the mechanism of action in the two mouse models is different.

Clinical studies related to the use of TAM for the treatment of DMD are still ongoing. In particular. Dirk Fischer and his team at the University Children’s Hospital of Basel are conducting an ongoing randomized, double-blind, placebo-controlled 48-week trial (Nagy et al., 2019) of oral TAM in DMD patients (TAMDMD, NCT03354039, EudraCT Number: 2017–004554–42). Patients are being enrolled at multiple European sites. At least 71 ambulant patients aged 6.5–12 years receiving stable treatment with glucocorticoids and 16–20 non-ambulant patients who are not treated with glucocorticoids are to be randomized to either TAM 20 mg/day or placebo for 48 weeks of treatment. At the end of the main trial, patients will be asked to enter an Open Label Extension (OLE) trial where all patients receive TAM. The purpose of the double-blind phase is to evaluate the effect of TAM on muscle function and force compared to placebo in ambulant and non-ambulant children with DMD. The therapeutic question is whether TAM can reduce the progression of the disease by at least 50% in ambulant patients under standard-of-care GCs and if it can decrease clinical decline for non-ambulant patients who do not receive GCs. Primary and secondary outcomes are measured with MFM analysis (Measurement of Motor Function), a reliable and validated assessment commonly used for neuromuscular disorders.

The first unpublished results shared in the clinical trial protocol (Nagy et al., 2019) are that TAM increases the levels of pro-inflammatory cytokines and growth factors associated with muscular regeneration and enhances muscle-purified mitochondria to buffer cytosolic calcium.

Information about dosage tolerance and long-term follow-up with TAM treatment in a relevant population are detailed in a study published by Derman et al., 2008. This study presents the outcomes of a follow-up of teenagers (11.5–14 years old) affected by mild gynecomastia. In this study, nine patients were treated with oral TAM at 10 mg twice daily and one with 20 mg twice daily. The treatment lasted an average of 5.7 months, but the follow-up assessments lasted a mean of 4.6 years with a monthly appointment for liver function tests and total blood count. Results showed that skeletal maturation was following its course within a physiological trend. Thrombocytopenia, significant changes in hormone levels, or liver function test abnormalities were not found, and TAM was very well tolerated overall by teenage patients.

## Conclusion and future development

In this review, substantial evidence demonstrates that TAM significantly impacts striated muscle regeneration and overcomes the lack of dystrophin in animal models.

Since the study of Dorchies et al. shows that very low levels of TAM and TAM metabolites are sufficient to cause significant therapeutic benefits in the dystrophic mouse, the possibility of a clinical application to patients with DMD is encouraging. It is possible that therapeutic TAM concentrations might be reached with lower than standard TAM doses, the safety of which has been established for more than 20 years.

The evaluation of TAM as a known orally active small-molecular weight compound with a well-characterized pharmacodynamic and safety profile presents compelling advantages over other therapeutic avenues. In particular, TAM might benefit patients with DMD within a short time and is much less costly than other options. Furthermore, compared to gene therapy, TAM treatment could offer access to all DMD patients regardless of their specific genetic profile.

Further clinical trials should be performed to provide relevant data and help the Duchenne research community highlight this treatment as a potential new standard of care for DMD patients.

## Abbreviations

6-MWT, 6-m Walking Test; AONs, Antisense Oligonucleotides; CPK or CK, Creatine Phosphokinase; DIA, Diaphragm; DHPR, Dihydropyridine Receptors; DMD, Duchenne Muscular Dystrophy; E2, Estradiol; ER, Estrogen Receptor; EMA; European Medicines Agency; GC, Glucocorticoids; GR, Glucocorticoid Receptor; MeSH, Medical Subject Headings; NF-kB, Nuclear Factor Kappa-light-chain-enhancer of Activated B Cells; NSAA, North-Star Assessment; Hsp, Heat-shock Protein; PICOS, Population Intervention Comparison Outcome Study type; RCT, Randomized Clinical Trial; SERM, Selective Estrogen Receptor Modulator; TAM, Tamoxifen; XLMTM, X-Linked Myotubular Myopathy.
